# Epidemiology and clinical characterization of invasive fungal infections in pediatric hemato-oncologic patients at a tertiary referral center in Northeastern Mexico

**DOI:** 10.1007/s00277-025-06655-w

**Published:** 2025-10-24

**Authors:** Denisse Natalie Vaquera-Aparicio, José Iván Castillo-Bejarano, Abiel Homero Mascareñas-de-los-Santos, Rogelio de Jesus Treviño, Paul Santiago Arcos-Viscarra, Marcela Lizeth Morales-López, Ana Sofía Guerrero-Delgado

**Affiliations:** 1 Department of Pediatrics / Infectious Diseases Service, Hospital Universitario “Dr. José Eleuterio González”, Francisco I. Madero Avenue, Mitras Centro, Universidad Autónoma de Nuevo León, Monterrey, 64460 ZC México; 2 Department of Microbiology, Universidad Autónoma de Nuevo León, Francisco I. Madero Avenue, Mitras Centro, Monterrey, 64460 ZC Mexico; 3 Hospital-Based Epidemiological Surveillance Unit, Christus Muguerza Alta Especialidad, Monterrey, 64060 Mexico

**Keywords:** Risk factors, Invasive fungal infections, Leukemia-Lymphoma, Mycoses, Neoplasms, Epidemiology

## Abstract

Invasive fungal infections (IFIs) are life-threatening complications in immunocompromised patients, particularly those with hematologic malignancies or undergoing transplantation. Despite advances in diagnostic methods and antifungal therapy, IFI-related mortality remains unacceptably high. Evidence from Latin America is scarce, limiting the understanding of regional epidemiology and outcomes. Our work aimed to analyze the epidemiological and clinical profiles of pediatric hemato-oncologic patients diagnosed with proven IFIs. We conducted a retrospective, cross-sectional study by reviewing medical records of patients diagnosed with proven IFIs according to the 2020 criteria of the European Organization for Research and Treatment of Cancer and the Mycoses Study Group, at the Hospital Universitario “Dr. José Eleuterio González” in northeast Mexico, between 2018 and 2024. Statistical analysis included descriptive and inferential methods. A p-value < 0.05 was considered statistically significant. Thirty-three patients were included (mean age 6 years; 54.5% male). Most (91%) were classified as high risk for IFIs, and acute lymphoblastic leukemia was the most frequent underlying malignancy (72.7%). Mold infections accounted for 69.7% of cases, mainly *Aspergillus spp.* and *Fusarium spp.*, while *Candida tropicalis* was the most common yeast. The sinonasal region was the predominant site of mold disease. Prophylaxis was administered in 69.7% of patients, most commonly with itraconazole. Amphotericin B was the primary therapeutic agent, alone or in combination with voriconazole, and 42.4% required surgical intervention. Overall mortality was 21.2%, higher in yeast infections (30%) compared with molds (17.4%). Intensive care unit admission was the only independent predictor of death (OR 35.4; *p =* 0.019). In pediatric hemato-oncologic patients, IFIs were predominantly associated with acute lymphoblastic leukemia, neutropenia, and induction chemotherapy. Mold infections accounted for most cases, and mortality remained high despite prophylaxis. These findings provide novel data from Latin America, where studies on pediatric IFIs are limited, and underscore the need for improved diagnostic and preventive strategies in high-risk populations.

## Introduction

 IFIs are considered opportunistic diseases, primarily affecting immunocompromised and critically ill patients, and are associated with high morbidity and mortality. The concept of IFIs has evolved, particularly with the advancement of diagnostic techniques. According to the 2020 consensus by the European Organization for Research and Treatment of Cancer and the Mycoses Study Group Education and Research Consortium (EORTC/MSG-ERC), the definition of IFIs depends on whether the causative organism is a yeast or a filamentous fungus. The methods employed varies, ranging from microscopic analysis to tissue or blood cultures, serology, or DNA amplification techniques. EORT/MSGERC classified IFIs as possible, probable, or proven (Table 1) [1].

**Table 1:** Diagnostic definitions of invasive fungal infections (EORTC/MSGERC 2020)

Category	Summary Definition	Criteria
Proven IFI	Direct evidence of an invasive fungal pathogen in a normally sterile site, with associated tissue damage.	•Histopathology showing hyphae or yeast cells invading tissue. •Positive culture from blood, cerebrospinal fluid, biopsy, or another sterile specimen. •Molecular identification (PCR/sequencing) in tissue with histological fungal elements also qualifies as proven IFI
Probable IFI	Presence of host factors **plus** compatible clinical/radiological findings **plus** mycological evidence.	•Host: prolonged neutropenia, hematopoietic stem cell transplant, intensive immunosuppression. •Clinical: pulmonary nodules, halo sign, cavitation, hepatosplenic lesions. •Mycology: positive galactomannan, β-D-glucan, PCR, or culture from bronchoalveolar lavage/airway sample.
Possible IFI	Presence of host factors **plus** suggestive clinical/radiological findings, without mycological confirmation.	•As above, but lacking microbiological or biomarker evidence.

*IFI* Invasive Fungal Infection

Proven IFIs due to molds require the presence of hyphae or yeast-like melanized forms observed in histopathological, cytopathological, or direct microscopic examination of a specimen obtained by needle aspiration or biopsy from a normally sterile site, accompanied by evidence of associated tissue damage. Alternatively, the recovery of a mold by culture from a sterile site, in the context of a compatible clinical syndrome, also qualifies. For yeasts, including *Candida spp*., proven infection requires the demonstration of yeasts or pseudohyphae in tissue with associated damage or a positive culture from a normally sterile site (e.g., blood, cerebrospinal fluid), consistent with clinical findings. In the case of *Cryptococcus spp*., a positive culture from a sterile site, detection of the organism in tissue, or a positive cryptococcal antigen test in cerebrospinal fluid or blood, in a compatible clinical context, is also considered proven [2].

Despite recent advances in the diagnosis and treatment of IFIs, the morbidity and mortality associated with this condition remain high, particularly in the absence of early diagnosis, which inevitably delays the initiation of antifungal therapy. These two factors are critical, as they represent key predictors of survival in IFI. Among the isolated pathogens, *Aspergillus spp*. and *Candida spp*. account for approximately 95% of all IFIs cases [3]. Invasive candidiasis is associated with mortality rates exceeding 30%, while invasive aspergillosis may surpass 50%, particularly among patients undergoing hematopoietic stem cell transplantation or intensive chemotherapy [2–4].

Antifungal prophylaxis has significantly altered the epidemiology of IFIs, and the incorporation of immunomodulatory agents into oncologic therapies has introduced new periods of heightened susceptibility. Historically, fluconazole has been widely used for prophylaxis in this population, resulting in a significant reduction in the incidence of invasive candidiasis. However, its limited spectrum—offering inconsistent coverage against non-*albicans Candida* species and no activity against *Aspergillus* or other molds—has prompted a shift in the paradigm. Current international guidelines now recommend broader-spectrum antifungal agents, such as newer triazoles with antifungal activity, to enhance prophylactic efficacy and reduce the burden of IFIs in high-risk pediatric patients [5, 6].

In recent years, a broader spectrum of fungal pathogens has emerged beyond *Candida albicans*, including non-*albicans Candida* species, *Trichosporon*, *Fusarium*, *Scedosporium*, and *Mucorales*. These pathogens often pose diagnostic challenges and display intrinsic or acquired resistance to antifungal agents, contributing to increased morbidity and mortality rates in affected patients [7, 8].

Documenting the incidence of IFIs in high-risk populations, such as the pediatric hemato-oncologic population, is crucial for informing the development of effective strategies for prevention, early diagnosis, and timely initiation of appropriate antifungal therapy. Generating robust epidemiological data may help reduce IFI-related mortality in pediatric patients. Moreover, the identification of prognostic factors remains crucial, as delayed catheter removal, late initiation of antifungal treatment, and early onset of infection have been linked to adverse clinical outcomes [9–11].

A deeper understanding of IFIs is crucial for informed clinical decision-making, as it directly impacts patient survival. In this context, evaluating the current epidemiological landscape of IFIs among pediatric hemato-oncologic patients becomes essential, particularly given the scarcity of local national data.

This study aimed to characterize the epidemiological profile and clinical outcomes of pediatric hemato-oncologic patients diagnosed with proven IFIs at a tertiary care referral hospital in northeastern Mexico.

## Materials and methods

An observational, retrospective, descriptive, and cross-sectional case series study was conducted from 2018 to 2024 at Hospital Universitario “Dr. José Eleuterio González” in Monterrey, Mexico. Our Institution is a third-level referral center and houses the “Centro Universitario Contra el Cáncer”, one of the largest oncology centers in Mexico.

Pediatric hemato-oncologic patients with proven IFIs, as defined by the 2020 EORTC/MSGERC criteria, were included in this study. Clinical and epidemiological information was extracted from medical records and consolidated into a structured database for subsequent analysis.

Given the retrospective design of the study, systematic environmental monitoring data (e.g., air sampling, fungal spore counts) were not available. Nevertheless, institutional records confirmed that no major construction activities took place in high-risk patient care areas during the study period. Hematology/oncology wards and operating rooms are equipped with high-efficiency particulate air (HEPA) filtration systems, and standard infection control measures—including strict hand hygiene protocols and protective isolation for severely immunocompromised patients—were consistently implemented. The EORTC/MSGERC classification defines IFIs as proven, probable, or possible, based on the degree of diagnostic certainty. Proven IFIs require histopathological evidence of fungal elements in tissue or positive culture from a sterile site. Probable IFI is established by the presence of predisposing host factors, compatible clinical and radiological findings, and mycological evidence, either through direct isolation or indirect detection of fungal biomarkers. Possible IFI is considered when host and clinical criteria are met in the absence of microbiological confirmation [1].

Our study was designed to include proven IFIs according to EORTC/MSGERC definitions. For pulmonary aspergillosis, invasive procedures such as lung biopsy are rarely feasible in the pediatric setting. Therefore, pulmonary cases were defined by the isolation of *Aspergillus spp.* from tracheal aspirates in combination with compatible clinical and radiological findings, together with a positive galactomannan assay. Although such cases would formally correspond to the “probable” category under EORTC/MSGERC criteria, they were considered clinically significant, managed as proven IFI in our institution, and retained in the analysis [1]. Patients included in the study were classified into two risk groups for IFIs according to criteria established in current clinical guidelines. Patients with fever and persistent neutropenia (>96 h) despite broad-spectrum antimicrobial therapy, a diagnosis of acute myeloid leukemia, relapse of acute leukemia, high-risk acute lymphoblastic leukemia, intensively myelosuppressive chemotherapy, prolonged neutropenia (>10 days), or use of high-dose corticosteroids were considered high risk. Patients who did not meet these criteria were classified as low risk.

Antifungal prophylaxis was prescribed in patients with prolonged or profound immunosuppression, at the discretion of the treating physicians and using standardized international guideline–recommended dosing adjusted for body weight or body surface area.

Patients with proven IFIs received systemic antifungal therapy following the same guideline-based principles, with a loading dose administered prior to maintenance therapy when indicated. For adolescents or patients whose body weight reached the threshold for adult regimens, dosing was adjusted to adult recommendations [12–17]. 

The duration of antifungal therapy was heterogeneous and individualized, depending on the site of infection, degree of immunosuppression, and clinical response.

Among the patients who received corticosteroids as part of their underlying disease treatment, exposure was heterogeneous. In the transplant setting, steroids were typically initiated at graft infusion and tapered within the first days, although some required longer courses for graft-related complications. In acute lymphoblastic leukemia, high-dose prednisone was administered during induction and subsequent phases, while in other hematologic conditions and solid tumors corticosteroids were not routinely used. For the purposes of analysis, clinically relevant exposure was defined as prednisone at high doses (≈ 2 mg/kg/day, ≈ 60 mg/m²/day) or when sustained for more than 21 days.

Descriptive statistics were used to summarize the data, including absolute frequencies and percentages for categorical variables, as well as means with standard deviations for continuous variables. Chi-square and Fisher’s exact tests were used for categorical comparisons, and an independent samples t-test for continuous variables. A univariate analysis was conducted to explore associations between clinical variables and mortality. Subsequently, a multivariate logistic regression model was applied to identify independent predictors of mortality, using clinically relevant variables with biological plausibility and/or significant trends in the univariate analysis. Variables included in the model were: fungal type (mold vs. yeast), presence of profound neutropenia (< 100 cells/µL), chemotherapy induction phase at the time of IFI diagnosis, and pediatric intensive care unit admission. Odds ratios (ORs) and 95% confidence intervals (CIs) were calculated. Statistical significance was defined as *p* < 0.05. All analyses were performed using SPSS version 25.0 (IBM Corp., Armonk, NY, USA).

## Results

A total of 33 pediatric hemato-oncologic patients were included, with a mean age of 6 years. Male patients accounted for 54.5% (*n =* 18/33). The mean age was lower among patients with yeast infections compared to those with mold infections (3.0 vs. 7.4 years, *p* < 0.001). Table 2 summarizes the demographic and clinical characteristics of pediatric hemato-oncologic patients with proven invasive fungal infections, stratified by fungal type.

**Table 2:** Demographic and clinical characteristics of pediatric Hemato-Oncologic patients with proven invasive fungal Infections, stratified by fungal type (Yeasts vs. Molds)

	Total (*N* = 33)*N* (%)			Yeasts (*N* = 10)*N* (%)		Molds (*N* = 23)*N* (%)				*P*		
Demographic data												
Female	15 (42.4)			5 (50)		10 (43.5)						
Male	18 (54.5)			5 (50)		13 (46.5)						
Age (years)	6.05 ± 4.03			2.96 ± 1.94		7.39 ± 3.99				*p* < 0.001		
Comorbidities, n (%)												
Hematologic neoplasms	28 (84.8)			5 (50)		23 (100)						
- ALL	24 (72.7)			5 (50)		19 82.6)						
- AML	3 (9.1)			0 (0)		3 (13)						
- Aplastic Anemia	1 (3)			0 (0)		1 (4.3)						
Solid tumors	5 (15.2)			5 (50)		0 (0)						
HSCT	6 (18.1%)			2 (20)		4 (17.4)						
Phase of treatment, n (%)												
Induction phase	26 (78.7)			6 (60)		20 (87)				*p* = 0.970		
Solid treatment	1 (3)			0 (0)		1 (4.3)						
Other treatment phases	5 (15.1)			4 (40)		2 (8.7)						
Risk factor n, (%)												
Prolonged neutropenia	24 (72.7)			4 (40)		20 (87)						
Profound neutropenia	21 (63.6)			3 (30)		18 (78.2)				*p* = 0.370		
Corticosteroid use	9 (27.2)			4 (40)		5 (21.7)						
Fungal Prophylaxis	23 (69.6)			4 (40)		19 (82.6)						
- Itraconazole	14 (42.4)			1 (10)		13(56.5)						
- Voriconazole	6 (18.1)			2 (20)		(17.3)						
- Fluconazole	3 (9)			2 (20)		(4.3)						
- Posaconazole	1(3)			0 (0)		1 (4.3)						
- Unknown	10 (30.3)			0 (0)		0 (0)						
CVC present	30 (91)			10 (100)		20 (87)				-		-
PICU stay	12 (36.3)			7 (70)		5 (21.7)				*p* = 0.019		
Dead	7 (21.2)			3 (30)		4 (17.4)						
Adjusted Odds Ratios for independent predictors of mortality												
Variable	OR [95% CI]					*p*						
Fungal type (mold vs. yeast)	3.80 [0.19–77.74]					*p* = 0.386						
Chemotherapy induction phase	1.05 [0.08–13.35]					*p* = 0.970						
Profound neutropenia	3.56 [0.22–57.16]					*p* = 0.370						
PICU stay	35.4 [1.82–688.4]					*p* = 0.019						

Data are presented as n (%) or mean ± SD. A *p* values were calculated using the t test or chi-square test, as appropriate. Odds ratios (OR) and 95% confidence intervals (CI) were calculated using multivariate logistic regression model. A *p* < 0.05 was considered statistically significant. Acute lymphoblastic leukemia (ALL); Acute myeloid leukemia (AML); Central Vascular Catheter (CVC), Pediatric Intensive Care Unit (PICU)

According to established risk stratification criteria, 91% (*n =* 30/33) of the cases were assigned to the high-risk group, while 9% (*n =* 3/33) were classified as low risk.

A total of 72.7% of patients (*n =* 24/33) had neutropenia defined as an absolute neutrophil count (ANC) < 500 cells/µL, with the same percentage experiencing prolonged neutropenia (> 10 days). Profound neutropenia was documented at the time of diagnosis in 63.6% (*n =* 21/33) of cases.

Most patients had underlying hematologic malignancies. Acute lymphoblastic leukemia was the most common diagnosis, present in 72.7% of cases (*n* = 24/33), followed by solid tumors in 15.2% (*n* = 5/33), acute myeloid leukemia in 9.1% (*n* = 3/33), and severe aplastic anemia in 3.0% (*n* = 1/33). In addition, 6 patients (18.2%) had undergone allogeneic HSCT, including 3 with ALL, 2 with AML, and 1 with aplastic anemia.

At the time of diagnosis, 81.2% (*n =* 26/32) of patients were undergoing induction chemotherapy, which represents the most immunosuppressive and infection-prone phase. Additionally, 15.6% (*n =* 5/32) were classified under other treatment phases, while only 3.1% (*n =* 1/32) were receiving a solid tumor-specific protocol. This distribution highlights the increased vulnerability to IFI during the early intensive treatment phases in pediatric oncology. Systemic corticosteroid use for more than three weeks was documented in 27.3% (*n =* 9/33) of patients.

Antifungal prophylaxis was administered in 69.7% (23/33) of patients. Among those who received prophylaxis (*n* = 23), agents used were itraconazole in 14 patients (60.9%), voriconazole in 6 (26.1%), fluconazole in 3 (13.0%), and posaconazole in 1 (4.3%). One patient received sequential azole prophylaxis, initially with itraconazole and subsequently switched to voriconazole due to drug availability. The remaining ten patients (30.3%) did not receive prophylaxis, five with solid tumors and five with hematologic malignancies. The most common fungal pathogens were *Candida spp*. and *Aspergillus spp*., accounting for 27.3% (*n =* 9/33) and 24.2% (*n =* 8/33) of cases, respectively. Among the *Candida* isolates, five were *C. tropicalis*, two were *C. albicans*, and two were *C. parapsilosis*. Only in one case was *Pneumocystis jirovecii* isolated.

Filamentous fungi accounted for 69.7% (*n =* 23/33) of all IFI cases in this cohort. The most frequent molds were *Aspergillus spp*. in 34.8% (*n =* 8/23) and *Fusarium spp*. in 34.8% (*n =* 8/23). Other molds included *Mucorales* such as *Apophysomyces spp*. and *Lichtheimia* (13.0%, *n* = 3/23), dematiaceous fungi (*Bipolaris spp*. and *Exserohilum spp*, (13.0%, *n =* 3/23), and hyaline hyphomycetes (8.7%, *n =* 2/23), which could only be classified at the group level, without species-level identification. The nasal and paranasal regions were the most affected anatomical sites among patients with mold infections, representing 56.5% (*n =* 13/23) of cases. This group included specimens from nasal mucosa biopsies, oral and palatal mucosa, all suggesting invasive sino-oronasal fungal disease. Pulmonary involvement was documented in four patients (17.4%), identified through tracheal aspirates positive for *Aspergillus spp.*, supported by compatible radiological findings, and further confirmed by a positive galactomannan assay. Cutaneous involvement confirmed by skin biopsy was also observed in 17.4% (*n* = 4/23) of cases. A single case of fungemia due to mold was confirmed via blood culture—specifically *Aspergillus spp*.—representing a rare but severe presentation of invasive mold infection, not related to vascular catheter use. Notably, this patient also had a concurrent skin biopsy yielding *Fusarium spp*., highlighting the possibility of mixed mold infections in severely immunocompromised hosts (Table 3).

**Table 3:**
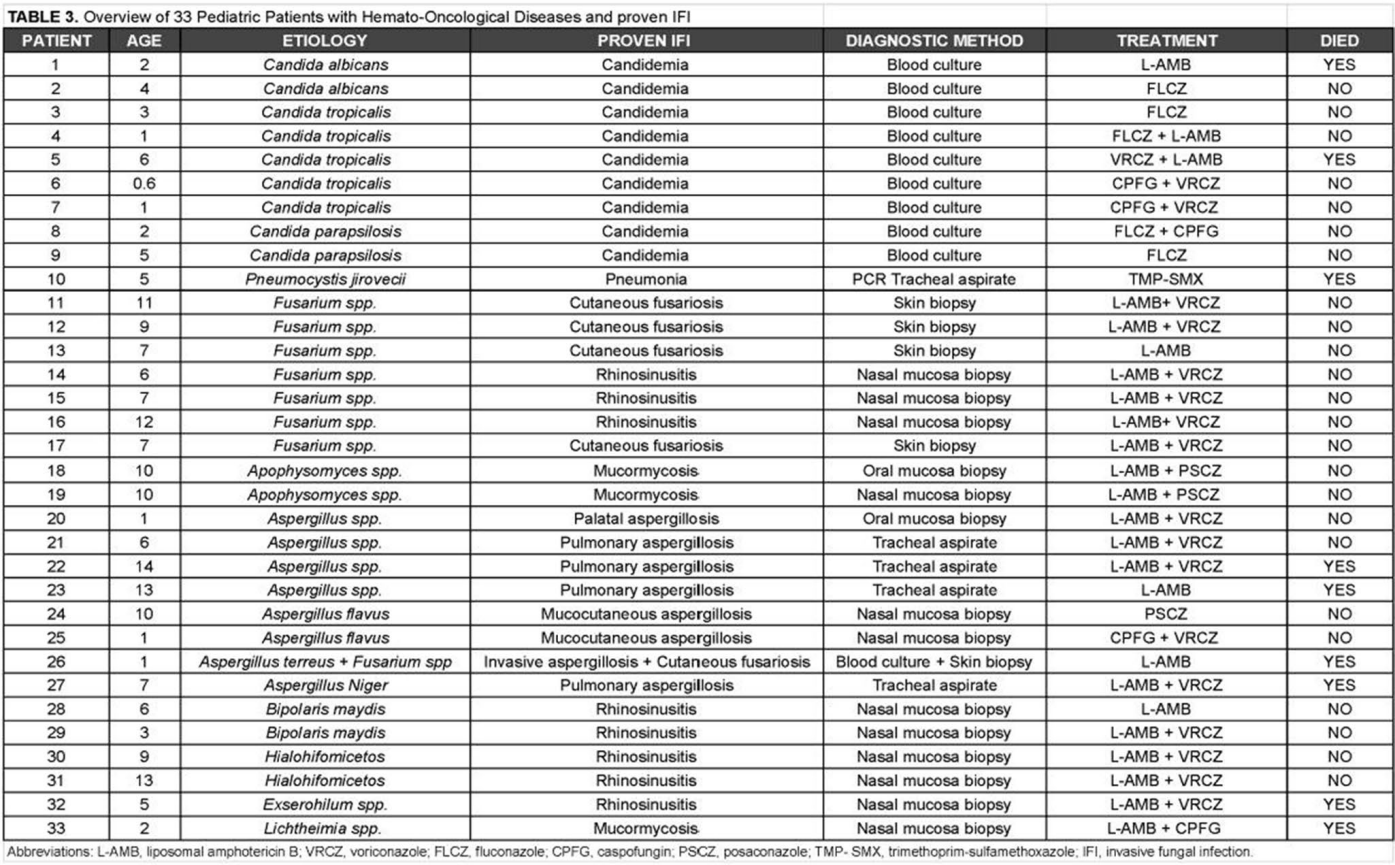
Overview of 33 Pediatric Patients with Hemato-Oncological Diseases and proven IFI

Regarding antifungal treatment, the most frequently administered combination was amphotericin B plus voriconazole, used as initial dual therapy in 48.5% (*n =* 16/33) of patients. Among those with mold infections, amphotericin B was used in 87.0% (*n =* 20/23) either alone or in combination, compared to voriconazole included in 69.6% (*n =* 16/23). In candidiasis, monotherapy predominated: amphotericin B and caspofungin were each used in 30.0% (*n =* 3/10) of cases.

Surgical intervention was required in 42.4% (*n* = 14/33) of patients, mainly those with sino-oronasal or palatal disease due to molds such as *Mucorales*,* Fusarium* spp., *and Aspergillus* spp., reflecting the need for aggressive source control in anatomically complex regions.

The overall mortality rate was 21.2% (*n =* 7/33). Among those with yeast infections, mortality reached 30.0% (*n =* 3/10), while in mold infections, it was slightly lower at 17.4% (*n =* 4/23).

A multivariate logistic regression model was conducted to identify independent predictors of mortality among pediatric patients with IFIs. The only variable significantly associated with mortality was intensive care unit (ICU) admission, with an OR of 35.4 (95% CI: 1.82–688.4, *p* = 0.019), indicating a markedly elevated risk of death among critically ill patients. Although neutropenia < 100 (OR 3.56, *p* = 0.376) and mold infection (OR 3.80, *p* = 0.386) showed numerical trends toward increased risk, they did not reach statistical significance. Similarly, receiving treatment during the induction phase of chemotherapy was not associated with increased mortality (OR 1.05, *p* = 0.970).

## Discussion

IFIs represent a significant cause of morbidity and mortality in hemato-oncologic patients, mainly attributable to their immunocompromised status. With the growing number of pediatric patients receiving chemotherapy and immunosuppressive therapies, IFIs must be considered a critical differential diagnosis in any febrile or infectious episode. This study provides a comprehensive evaluation of the clinical epidemiology of IFIs in pediatric hemato-oncologic patients, reinforcing the importance of early recognition and targeted management to improve patient outcomes.

Current clinical guidelines define high risk for IFIs as those patients with persistent fever and neutropenia for more than 96 h despite broad-spectrum antimicrobial therapy; patients with underlying hematologic malignancies such as acute myeloid leukemia, relapsed acute leukemia, or high-risk acute lymphoblastic leukemia; individuals receiving highly myelosuppressive chemotherapy for other neoplasms; those expected to experience prolonged neutropenia (>10 days); and patients undergoing high-dose corticosteroid therapy. Moreover, individual susceptibility to IFIs has been linked to host-related factors, including genetic polymorphisms in innate immune receptors such as TLR2, TLR4, dectin-1, and pentraxin-3 [18]. In summary, IFIs risk is determined by host-related factors and the underlying onco-hematologic disease. Patients who do not meet these criteria are classified as low risk. Among the patients analyzed 91% of IFIs cases occurred in high-risk patients, reinforcing the critical role of profound immunosuppression in the development of these infections. This distribution is consistent with that reported by Pagano et al., Gutierrez et al., and Fisher et al., who documented a higher incidence of IFIs in pediatric populations with acute leukemias and prolonged neutropenia [19–21].

Neutropenia, particularly in terms of its severity and duration, plays a critical role in the development of IFIs. This association is attributed to impaired innate immune responses, as neutrophils serve as a frontline defense against fungal pathogens through phagocytosis and the release of reactive oxygen species and antimicrobial peptides [3, 10]. In our cohort, neutropenia emerged as a predominant risk factor: 72.7% of patients (*n =* 24/33) had absolute neutrophil counts below 500 cells/µL, and an equal proportion experienced prolonged neutropenia lasting more than 10 days. Moreover, 63.6% (*n =* 21/33) exhibited profound neutropenia at the time of IFI diagnosis. These findings are consistent with previous studies that underscore the depth and duration of neutropenia as critical determinants in the development and severity of invasive fungal infections (IFIs) among immunosuppressed pediatric patients [3, 18, 22]. Notably, a study conducted in the Asian region reported that neutropenia lasting more than 7 days was observed in up to 93.9% of cases [22]. However, comparable data from studies conducted in our region remain limited, highlighting the need for further investigation in similar epidemiological contexts.

Regarding underlying conditions, B-cell acute lymphocytic leukemia was the most frequently associated malignancy, found in 71% of patients. This contrasts with other reports where acute myeloid leukemia is cited as the leading hematologic disease associated with IFI [23]. Similarly, the incidence of solid tumors (14%) in this patient population is comparable to figures reported in prior studies (12–15%) [24].

In the present study, most patients had underlying hematologic malignancies. Acute lymphoblastic leukemia was the most prevalent diagnosis, accounting for 72.7% (*n =* 24/33) of cases, followed by solid tumors (15.2%, *n =* 5/33). This distribution underscores the predominance of acute lymphoblastic leukemia within pediatric hemato-oncologic populations and its strong association with intensive immunosuppressive therapies, which elevate the risk for IFI. Notably, acute myeloid leukemia has been identified as a significant risk factor for the development of IFIs, with studies indicating a higher incidence compared to other malignancies [3, 23]. For instance, a multicenter cohort study reported that children with acute myeloid leukemia had a proven/probable IFI prevalence of 10.3% during primary therapy, highlighting the need for vigilant monitoring and prophylactic strategies in this high-risk group [24].

In our cohort, most IFIs were diagnosed during the induction phase of chemotherapy, with 81.2% (*n =* 26/32) of cases occurring in this period. This phase is characterized by intensive immunosuppressive therapy, leading to profound and prolonged neutropenia. These findings underscore the heightened vulnerability during the early intensive treatment phases of pediatric oncology.

This observation aligns with existing literature. A study by Yigit et al. reported that the requirements for antifungal therapy increased with the intensity of chemotherapy across all leukemia risk groups. Notably, intermediate-risk patients required more antifungal therapy during induction, whereas high-risk patients needed it throughout both the induction and consolidation phases. This suggests that the induction phase, particularly in intermediate-risk patients, is associated with a significant risk of IFIs [25].

Furthermore, a prospective multicenter surveillance study by Castagnola et al. found that 73% of fungal infection episodes in children with cancer occurred following aggressive chemotherapy, with neutropenia present in 77% of cases. The study highlighted that severe and prolonged neutropenia during intensive chemotherapy phases, such as induction, is associated with an increased risk of fungal infections [26]. These findings collectively emphasize the critical need for vigilant monitoring and potential prophylactic strategies during the induction phase of chemotherapy in pediatric oncology patients to mitigate the risk of IFIs.

Systemic corticosteroids are a significant factor in the development of IFI, particularly in patients with hematological-oncological diseases, where these drugs are part of routine therapeutic management. Their administration has been associated with significant immunosuppression, compromising both innate and adaptive immunity, and inducing dysregulation of immunological mechanisms [3, 27]. Current evidence suggests that the dose and duration of corticosteroid treatment significantly influence the risk of developing IFIs, with prolonged use for more than 21 days being a recognized predisposing factor [28, 29]. In a previous study, it was reported that up to 50% of patients with a hematological-oncological diagnosis and proven IFI had received systemic steroids for more than three weeks [30]. Compared to our results, this factor was documented in 27.3% of cases, which could be attributed to variations in the intensity of immunosuppressive treatment, host characteristics, or possible limitations in clinical records. Despite this discrepancy, our findings underscore the importance of rigorously evaluating the use of systemic corticosteroids, especially considering their potential impact on the development of IFIs. It is essential to tailor the dose and duration of treatment to the individual host’s condition, always prioritizing the use of the lowest effective dose for the shortest possible duration to minimize the risk of infection [27].

In our cohort, antifungal prophylaxis was administered in 69.7% of patients, while the remaining ten did not receive it—five with solid tumors, for whom prophylaxis is not routinely indicated according to established pediatric oncology guidelines, and five with hematologic malignancies who did not fulfill the clinical criteria for initiation prior to admission [31]. Nevertheless, proven IFIs developed both in patients with and without prophylaxis, suggesting that prophylaxis was insufficient rather than fully protective. A key contextual factor is that in our center voriconazole, the preferred agent in many high-income countries, is largely restricted to allogeneic HSCT recipients due to financial constraints, limiting its broader use in other high-risk populations. As a result, most patients received itraconazole or fluconazole, which may provide less effective protection against invasive mold infections. These resource-based limitations should be considered when comparing IFI rates across institutions. Nonetheless, the use of oral azoles in our cohort remains aligned with current recommendations for pediatric patients at high risk for IFIs, such as those with hematologic malignancies [32, 33].

Despite the availability of antifungal agents, a proportion of IFIs still occurs during prophylaxis, potentially due to antifungal resistance, suboptimal drug exposure, or non-adherence to treatment [34]. These findings underscore the importance of implementing individualized prophylactic strategies tailored to patient-specific risk profiles, underlying malignancies, and therapeutic regimens. Furthermore, appropriate documentation and monitoring are crucial for evaluating the real-world effectiveness of prophylaxis in clinical practice. Although antifungal stewardship indicators were not assessed in this retrospective study, we recognize this as an area of opportunity under development in our institution. Previous reports have shown that antifungal stewardship programs in pediatric hemato-oncology and HSCT settings can improve the rational use of antifungals, optimize diagnostics, and potentially reduce IFI-related morbidity and costs [35].

In this study, *Candida spp*. and *Aspergillus spp*. were the most frequently isolated fungal pathogens, accounting for 27.3% and 24.2% of the IFIs proven, respectively. These findings are consistent with those previously reported in other pediatric series, where *Candida spp*. is often the predominant agent, especially in settings with prolonged immunosuppression or the use of vascular devices [5, 13]. Consistently, in our study, all *Candida spp*. bloodstream infections were associated with the presence of intravascular devices, with non-albicans species, particularly *C. tropicalis*, being the most frequently identified. The documentation of *Aspergillus* fungemia in our series represents an uncommon but clinically relevant manifestation of invasive mold disease. Blood cultures are notoriously insensitive for molds, with reported sensitivities of only 1–5%, in contrast to 50–95% for *Candida* spp. These limitations reinforce the role of complementary diagnostic strategies—such as galactomannan, β-D-glucan assays, and molecular methods—in improving the early recognition of invasive mold infections [36]. 

A mold infection was reported in 69.7% of cases; these accounted for the majority of patients in our series, reinforcing their growing role as relevant etiologic agents in onco-hematological patients. *Aspergillus spp.* and *Fusarium spp.* (34.8%) were the most common molds. A finding consistent with that reported in larger studies, where *Aspergillus spp*. represents between 58% and 90% of mold infections [2, 19]. The finding of fusariosis in almost a third of mold infections in our series is particularly relevant considering that this agent has been classified as an emerging pathogen with high mortality and limited therapeutic response to conventional antifungals [2].

Furthermore, the detection of *Mucorales*, dematiaceous fungi, and hyaline hyphomycetes in our series reinforces the findings of previous studies, which highlight the increasing role of rare fungal infections in immunocompromised patients [34]. Compared to a study conducted in Turkey, which reported rare fungal infections in 4.8% of all IFI episodes with an increased proportion of 31.5% in proven cases, our data suggest a higher frequency of these organisms, even when considering the total number of IFI episodes [37]. This discrepancy may reflect differences in diagnostic practices, host characteristics, or epidemiological factors, but it further supports the notion that rare molds are becoming increasingly significant and must be actively considered in high-risk patients. These agents, although uncommon, present significant diagnostic and therapeutic challenges and are associated with adverse clinical outcomes, especially in the absence of early diagnosis and appropriate antifungal or surgical intervention.

*Pneumocystis jirovecii* infection remains a potentially serious complication in this patient population; however, its epidemiology has not been comprehensively characterized [19]. Recent data suggest a significant decline in incidence, with an international study reporting a cumulative rate as low as 0.09% over a three-year period [38]. In our research, *Pneumocystis jirovecii* was isolated in only one case, further supporting the observed low prevalence of this pathogen.

The predominance of upper airway and mucosal sites contrasts with classical presentations reported in studies emphasizing pulmonary forms [39, 40], and may reflect local diagnostic patterns, tissue-invasive behavior of specific pathogens, or earlier detection through mucosal surveillance in immunocompromised patients.

Several studies have reported mortality rates associated with IFIs ranging from 21% to 25%, findings that align with the overall mortality rate observed in our cohort (21.2%) [9, 41]. Interestingly, a higher mortality rate was noted among patients with yeast infections (30.0%) compared to those with mold infections (17.4%). This observation contrasts with the general trend described in the literature, where mold infections, particularly those caused by *Aspergillus* and *Fusarium spp*., are typically associated with higher mortality rates. However, it is essential to emphasize that all *Candida spp*. infections in our study presented as candidemia, a severe manifestation known to reach mortality rates of 30–40%, especially in immunocompromised hosts [41]. Moreover, most fatal cases in the yeast group involved patients with multiple comorbidities and complex clinical conditions, complicating the attribution of mortality exclusively to the fungal infection. These findings suggest a multifactorial outcome in which candidemia likely acted as a significant contributor within a broader context of critical illness. We hypothesize that the relatively lower mortality observed in proven IFI cases may be attributed to our proactive diagnostic approach, including intensive efforts to identify etiological agents and confirm infections, which allowed for targeted antifungal therapy. Furthermore, the early initiation of aggressive antifungal treatment upon clinical suspicion (probable IFI) and the use of a multidisciplinary management strategy—including surgical, ENT, and other specialty consultations—likely contributed to improved patient outcomes.

In our multivariate analysis, admission to the ICU was the only independent predictor of mortality with an OR of 35.4, underscoring the significantly elevated risk of death in critically ill patients. This is in line with a previous study, which consistently identified ICU admission as a significant risk factor for mortality in IFI. Reported overall fatality rates for IFIs range between 10% and 25% but can approach or exceed 50% among patients requiring intensive care unit support [42]. These findings underscore the importance of early diagnosis and intervention in preventing clinical deterioration and reducing the need for ICU admission, which is associated with poorer outcomes. Interestingly, treatment during the induction phase of chemotherapy was not associated with increased mortality in our analysis OR 1.05, differing from reports that describe this phase as particularly vulnerable due to intense immunosuppression and risk of infection [42]. Although we observed numerical trends suggesting increased risk associated with profound neutropenia and mold infections, these associations did not reach statistical significance in our cohort. These discrepancies may reflect differences in sample size, patient characteristics, supportive care measures, or timing of antifungal intervention across studies.

### Limitations

This study has several limitations. First, some isolates could only be identified to the genus level (*Aspergillus* spp., *Fusarium* spp., *Bipolaris* spp., *Exserohilum* spp.), and hyaline hyphomycetes could not be further characterized, reducing epidemiological precision and precluding species-specific conclusions. Second, the diagnosis of pulmonary aspergillosis relied on tracheal aspirates, radiological findings, and galactomannan rather than histopathology. Although this approach does not strictly align with EORTC/MSGERC definitions and may limit comparability with other series, it reflects real-world pediatric practice where invasive procedures such as lung biopsy are rarely feasible. Third, the retrospective design makes the study dependent on the accuracy of medical records and limits causal inference. Finally, although the sample size was relatively small, this reflects the rarity of proven IFIs in pediatric hemato-oncologic patients and underscores the value of documenting this cohort. Despite these limitations, the study provides meaningful insight into the epidemiology, clinical presentation, and outcomes of pediatric IFIs in Latin America and highlights the need for larger multicenter studies.

## Conclusion

In our work, IFIs were predominantly associated with profound and prolonged neutropenia, induction chemotherapy, and underlying hematologic malignancies, particularly acute lymphoblastic leukemia.

The etiological diversity observed reflects the evolving epidemiology of IFIs in this vulnerable population, encompassing not only classical pathogens such as *Aspergillus* spp. and *Candida* spp., but also an increasing number of emerging fungal species. Despite intensive antifungal therapy and surgical interventions, mortality remained high, with ICU admission identified as the sole independent predictor of death, underscoring the severe prognosis associated with critical illness.

Although antifungal prophylaxis was widely implemented, the persistence of IFIs and the inadequate documentation of prophylactic regimens in a substantial proportion of patients highlight the need to strengthen preventive strategies, ensure therapeutic adherence, and tailor interventions according to individual risk profiles and local epidemiological patterns. These findings reinforce the importance of active microbiological surveillance and the dynamic adaptation of antifungal prophylaxis policies to emerging threats.

These findings emphasize the need for early diagnostic strategies and prompt therapeutic interventions, as diagnostic delays markedly impact outcomes, particularly given the nonspecific clinical manifestations of IFIs in immunocompromised hosts. Maintaining a low threshold for clinical suspicion, utilizing early diagnostic imaging, biomarker assays, invasive sampling, and initiating targeted antifungal therapy without delay are pivotal measures to improve survival rates. Optimizing early diagnostic capabilities through risk stratification and preemptive or empirical antifungal strategies will be crucial in mitigating the burden of IFIs in this high-risk population.

## Data Availability

The data that support the findings of this study are not publicly available due to the inclusion of sensitive and identifiable patient information. Access to the data may be granted upon reasonable request. Requests should be directed to the corresponding author.
